# Computed Tomography-Based Assessment of Sarcopenia and Disease Progression in Pancreatic Ductal Adenocarcinoma: A Radiomics and Machine Learning Approach

**DOI:** 10.14740/gr2132

**Published:** 2026-06-16

**Authors:** Jean-Philippe R. Krieg, Luisa Gallee, Daniel Wolf, Konstantin Muller, Michael Goetz, Thomas J. Ettrich, Thomas Seufferlein, Christopher Kloth, Meinrad Beer, Daniel Vogele

**Affiliations:** a Department of Diagnostic and Interventional Radiology, University Hospital Ulm, 89081 Ulm, Germany; b XAIRAD—Artificial Intelligence in Experimental Radiology, University Hospital Ulm, 89081 Ulm, Germany; c Institute for Media Informatics, Visual Computing Group, University of Ulm, Germany; d i2SouI—Innovative Imaging in Surgical Oncology Ulm, University Hospital Ulm, 89081 Ulm, Germany; e Comprehensive Cancer Center Ulm (CCCU), University Hospital Ulm, 89081 Ulm, Germany; f Department of Internal Medicine I, University Hospital Ulm, 89081 Ulm, Germany

**Keywords:** Sarcopenia, Pancreatic cancer, CT radiomics, Machine learning, Tumor progression

## Abstract

**Background:**

Sarcopenia is a known negative prognostic factor in oncology and is frequently observed in patients with pancreatic ductal adenocarcinoma (PDAC). Computed tomography (CT) enables longitudinal muscle assessment and may provide additional prognostic information. This study aims to assess their association with prognosis in pancreatic cancer and explore the diagnostic and prognostic value of CT radiomics.

**Methods:**

A retrospective single-center study included 62 patients with primary PDAC who underwent at least three abdominal CT scans: baseline (t0), 3 months (t1), 6 months (t2), and, in 35 patients, 12 months (t3). CT-based sarcopenia was assessed using the psoas muscle index (PMI) based on reference cutoffs and cohort-specific sex-specific quartiles. Skeletal muscles at the L3 level were semi-automatically segmented. Radiomic features of the psoas were extracted and analyzed using k-nearest neighbor, decision tree, and random forest models. Prognostic relevance was evaluated using logistic regression and feature selection via least absolute shrinkage and selection operator (LASSO) regression. Tumor progression was assessed radiologically according to RECIST 1.1 criteria.

**Results:**

CT-based sarcopenia prevalence was 45.3% using reference-based PMI thresholds. PMI declined significantly from baseline to t1 and remained stable thereafter, with women exhibiting consistently lower values. Outcome analysis showed a higher proportion of disease progression at t1 in sarcopenic patients using reference cutoffs, whereas cohort-specific quartiles demonstrated no consistent differences. Random forest models predicted sarcopenia with up to 0.73 accuracy and receiver operating characteristics area under the curve (ROC-AUC) of 0.81. LASSO regression identified the psoas short axis and cross-sectional area as the most informative features. Logistic regression using baseline radiomic features predicted disease progression status at 12 months with 0.85 accuracy, weighted F_1_ 0.841, and AUC 0.823. Interobserver agreement for psoas measurements was high (r = 0.86).

**Conclusion:**

Longitudinal CT-based assessment of PMI demonstrates progressive sarcopenia within the studied PDAC cohort, with sex-specific declines. Radiomic analysis of skeletal muscle provides complementary information and predictive insights, highlighting their potential to enhance the characterization of muscle status and its association with disease course in patients able to undergo repeated imaging.

## Introduction

### Sarcopenia and clinical relevance in oncology

Sarcopenia is a progressive, generalized loss of muscle mass and strength. According to the European Working Group on Sarcopenia in Older People (EWGSOP), it can be classified as primary (age-related) or secondary, with the latter arising from systemic disease, chronic inflammation, or organ dysfunction [1]. While often associated with aging, secondary sarcopenia is common in younger and critically ill patients, including those with malignancies. Cancer-associated sarcopenia has been reported across a wide range of tumor types and age groups, including children and adolescents [2–4]. In oncology, sarcopenia is of significant clinical importance, as it is associated with diminished quality of life, heightened treatment-related toxicity, and adverse clinical outcomes. For example, Nipp et al reported high sarcopenia prevalence in patients with newly diagnosed, incurable cancer, accompanied by increased depressive symptoms and impaired quality of life, while Pamoukdjian et al found that nearly 39% of cancer patients exhibited sarcopenia prior to treatment initiation [5, 6]. These findings highlight sarcopenia’s potential as a prognostic biomarker influencing treatment tolerance and disease course [7–10].

### Sarcopenia in pancreatic tumors

Pancreatic tumors are strongly associated with sarcopenia. A systematic review by Haiducu et al reported a prevalence of approximately 45% in patients with pancreatic cancer [11]. Pancreatic cancer remains one of the most lethal malignancies, with a 5-year survival rate of only 10% in Germany in 2019. This poor prognosis is largely attributable to late-stage diagnosis, with nearly half of patients presenting with metastatic disease at the time of detection [12]. Curative treatment options are limited: only ∼15% of patients are eligible for surgical resection, and even after complete (R0) resection, median survival ranges 17–28 months, decreasing to 8–22 months for incomplete resections (R1/R2) [13]. While tumor-specific factors such as TNM stage, tumor biology, and resection status are established prognostic determinants, patient-related factors—including nutritional status, muscle mass, and physical resilience—are increasingly recognized as critical contributors to outcomes [14, 15]. Sarcopenia has emerged as a potential predictor of clinical outcomes in pancreatic ductal adenocarcinoma (PDAC). Reduced skeletal muscle mass or quality is associated with poorer overall survival, increased chemotherapy-related toxicity, and diminished treatment response [7, 16]. Peng et al first identified sarcopenia as a strong predictor of survival following pancreatic tumor surgery. Subsequent studies and meta-analyses, including those by Shachar et al and Kim et al, have confirmed the association of skeletal muscle index (SMI) and skeletal muscle density (SMD) with adverse outcomes in PDAC [7, 17, 18].

### Radiomics as a tool for sarcopenia assessment and prognostication

Radiomics refers to the extraction of quantitative imaging features beyond visual assessment, including shape, texture, and intensity-based characteristics that reflect tissue heterogeneity [19, 20]. These high-dimensional datasets can be analyzed using artificial intelligence (AI) and machine learning algorithms to identify patterns predictive of disease progression [21–24]. Beyond tumor characterization, radiomics has also been applied to skeletal muscle, enabling the assessment of muscle quality and composition in addition to muscle mass. Recent studies, including our own, demonstrated that computed tomography (CT)-based radiomics combined with machine learning can identify sarcopenia and predict clinical outcomes, offering a complementary tool to conventional muscle measurements [25].

### Purpose of the study

This retrospective single-center study aimed to examine the association between sarcopenia and clinical outcomes in PDAC. Specifically, we evaluated whether sarcopenia predicts disease progression at three predefined follow-up time points and compared two radiological definitions of sarcopenia. Additionally, we investigated the potential of CT radiomics combined with machine learning to enhance sarcopenia detection and prognostication. In this study, the term “sarcopenia” is used as a pragmatic, imaging-based term referring to CT-derived measures of reduced muscle mass and radiomic surrogates of muscle quality, as functional parameters such as muscle strength and physical performance are not available in this retrospective cohort. We hypothesized that 1) sarcopenia detected on routine staging CT scans influences disease progression in PDAC, and 2) CT radiomics of skeletal muscle combined with machine learning can reliably identify sarcopenia and predict tumor progression.

## Materials and Methods

The study was conducted in accordance with the Declaration of Helsinki, and approved by the Institutional Ethics Committee (Protocol Code 219/2020, Date of Approval 09/14/2020). The study was HIPAA compliant.

### Patient recruitment

Patients consecutively treated from 2015 to 2019 for primary PDAC were considered for inclusion. Eligible patients were required to have at least three evaluable abdominal CT scans at distinct time points: baseline prior to treatment (t0), and follow-ups at 3 months (t1), 6 months (t2), and optionally 12 months (t3), with allowable deviations ≤ 1 month. Patients were excluded if imaging was limited to other modalities (magnetic resonance imaging (MRI), ultrasound) or if CT quality was insufficient.

Demographic, clinical, and imaging data were collected from electronic medical records, picture archiving and communication system (PACS), and radiology information system (RIS). A total of 378 contrast-enhanced portal venous phase CT datasets were included. Scans were acquired on a 256-channel Philips Brilliance iCT (Philips, Eindhoven, Netherlands) or 128-channel Siemens Somatom Definition AS+ (Siemens Healthineers, Erlangen, Germany) using standard parameters (120 kV, automatic tube current modulation, matrix 512 × 512, in-plane resolution 0.62–0.86 mm^2^) with intravenous Ultravist^®^ 370 (Bayer Schering Pharma, Berlin, Germany) at a weight-matched dose (1.1 mL/kg) followed by 60 mL saline, 90 s delay. Axial reconstructions with 3–5 mm slice thickness were used.

Time points were defined relative to baseline: t0 (diagnosis), t1 (3 months), t2 (6 months), and t3 (12 months, available in 35 patients). Mean intervals were 86.2 ± 18.9, 174.9 ± 31.3, and 343.9 ± 35.2 days for t1, t2, and t3, respectively. Tumor progression as primary endpoint was assessed according to RECIST 1.1 by a board-certified radiologist with 11 years’ experience [26]. Disease progression reflects any RECIST-defined progressive disease (PD) with either any new lesions or at least 20% relative and 5 mm absolute increase of sum of diameters (SOD) of target lesions compared to smallest SOD ever recorded. Patients who died before follow-up imaging were excluded.

### Assessment of sarcopenia

CT images were analyzed using Mint Lesion™ (v3.8.4, mint Medical GmbH, Heidelberg, Germany). Muscle segmentation was performed at the L3 vertebral level on the lowest slice where the right transverse process was fully visible. The region of interest included the psoas major, quadratus lumborum, erector spinae, and rectus abdominis. Semi-automatic segmentation was applied.

The psoas muscle index (PMI) was calculated as psoas area (cm^2^) divided by height squared (m^2^). Only the right psoas major was analyzed as previous research, including a UK biobank study involving 5,000 participants, has shown negligible differences between the left and right psoas muscles [2, 4, 27]. Sarcopenia was defined using two methods: 1) reference-based cutoffs from Bahat et al [28]: 3.6 cm^2^/m^2^ for women, 5.4 cm^2^/m^2^ for men; and 2) cohort-specific lowest sex-specific quartiles [7, 28–33] (Table 1).

**Table 1:** Cohort-Derived Quartile Thresholds for Psoas Muscle Index (PMI) at Different Time Points for the Present Study Cohort

Time points	PMI in cm^2^/m^2^, female	PMI in cm^2^/m^2^, male
t0	3.6	5.0
t1	3.4	4.6
t2	3.1	4.7
t3	3.4	4.5

### Radiomics and machine learning

Radiomic features were evaluated based on their distinct grayscale patterns in accordance with the Image Biomarker Standardization Initiative (IBSI) guidelines and previously published settings [23, 25]. Radiomic features were obtained through the discretization method of fixed bin number (FBN), utilizing a bin amount of 32. To accentuate regions of rapid intensity change, a Laplacian of Gaussian (LoG) filter was employed as a preprocessing step, employing a sigma value of 2. The aggregation method used for matrix computation was “3D average directions.” The radiomic features were extracted with a resample filter and voxel resampling size (X, Y, Z) set at 1 × 1 × 1 mm^3^. Additionally, a second-order distance of 1 was considered. Intensity calculation for radiomic features was restricted within a specific range using a threshold filter set at 0. An overview of the extraction parameters is shown in Table 2. A total of 76 features were extracted from the psoas muscle. These included descriptors of size and shape, first-order statistics characterizing the distribution of voxel intensities within the region of interest, and texture features derived from the gray-level co-occurrence matrix.

**Table 2:** Settings of the Radiomics Feature Extraction

Setting	Determination
Bin method	FBN
Bin amount	32
LoG filter	0
LoG sigma	2
Matrix aggregation	3D average
Method	Directions
Resample filter	1
Resample spacing X	1
Resample spacing Y	1
Resample spacing Z	1
Second-order distance	1
Threshold filter	0

FBN: fixed bin number; LoG: Laplacian of Gaussian.

Data were pseudonymized and processed using standardized software [20]. Machine learning models included k-nearest neighbor (KNN), decision tree, and random forest (RF). In addition, a so-called least absolute shrinkage and selection operator (LASSO) regression was conducted with α = 0.24 to identify the most relevant predictor variables. A stratified split was used with exact train and test split proportions of 63.0% training and 37.0% test data sets with a balanced proportion of patients with PD of 61.8% and 60.0%, respectively. The samples used for feature selection and for the subsequent training of the classification model were drawn exclusively from the training dataset. The test data are used exclusively during the inference phase to calculate the test accuracy of the classification model. Experiments on outcome were repeated using random patient splits. Class imbalance was taken into account when assigning data to the training and testing splits. These were stratified according to class values, thereby ensuring that the distribution of classes is identical in both the training and testing splits. Predictive performance was evaluated using accuracy and receiver operating characteristics area under the curve (ROC-AUC) metrics.

### Statistical analysis

The primary statistical analyses were conducted using IBM SPSS Statistics (IBM Corporation, New York, USA, Version 29). Descriptive statistics included frequencies, percentages, means, and medians. Inferential analyses employed repeated measures analysis of variance (ANOVA) with Bonferroni correction, χ^2^-test, Fisher’s exact test, Mann–Whitney U-test, and correlation analyses as appropriate. Effect sizes were reported using partial η^2^ for ANOVA and *t*-tests, and Cramer’s V for categorical analyses. Significance was set at α = 0.05. Interobserver agreement was evaluated using the Pearson correlation coefficient.

Machine learning analyses were conducted in Python 3.11.5 using matplotlib, numpy, pandas, sklearn, and imblearn libraries [34–38]. Predictive models were trained and validated to assess sarcopenia and outcome prediction. Model performance was evaluated using multiple metrics: accuracy, which measures the proportion of correct predictions; weighted F_1_-score, which represents the harmonic mean of precision and recall and accounts for class imbalance; and AUC, which reflects the model’s overall discriminative ability. To ensure robustness and reproducibility, all models were trained and tested repeatedly using random patient splits, and performance metrics were averaged across runs.

## Results

**Figure 1:**
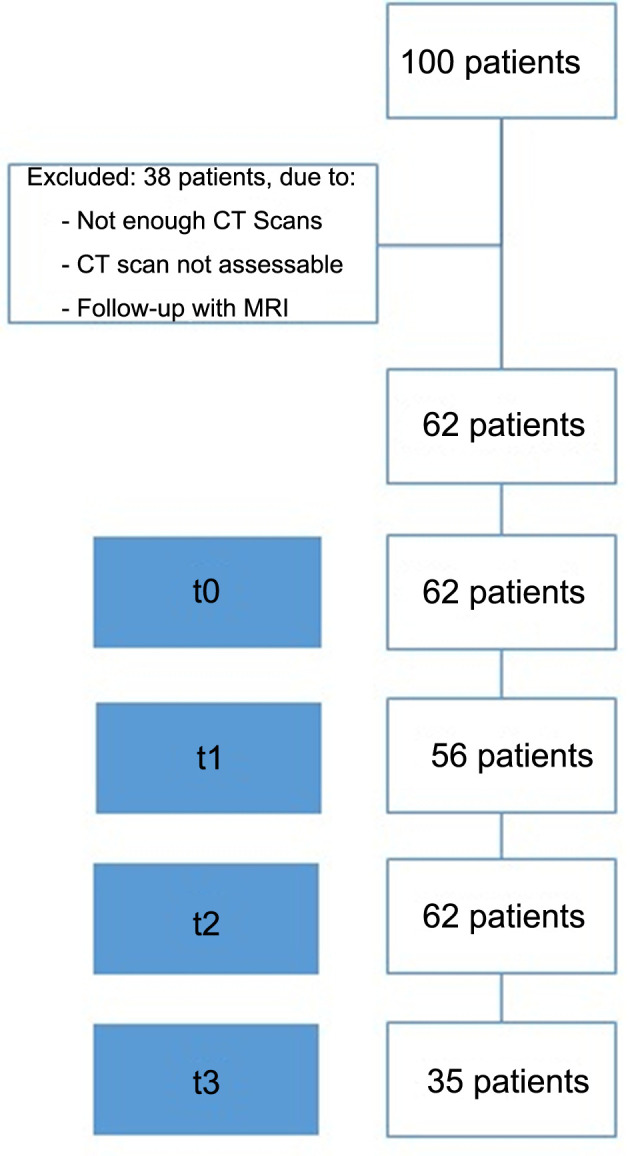
Patient recruitment for the study cohort.

Of the 100 patients initially screened, 62 met inclusion criteria and were included in the analysis (Fig. 1). The cohort was predominantly advanced stage, with Union Internationale Contre le Cancer (UICC) stages distributed as follows: IA, 4.8%; IB, 3.2%; IIB, 25.8%; III, 8.1%; and IV, 53.2%. During the disease course at t1, six of the 62 included patients did not have evaluable CT scans. At t2, CT scans were available for all 62 initially included patients. At t3, only 35 patients remained for analysis due to death (n = 21) or inadequate imaging (n = 6) (Fig. 1). All baseline and follow-up CT scans were evaluated and patient outcomes were defined as disease progression based on follow-up CT scans and categorized on an ordinal scale from 1 to 3. According to this classification, 59.7% of patients were in category 1, 29.0% in category 2, and 11.3% in category 3. Baseline demographic and clinical characteristics are summarized in Tables 3–5.

**Table 3:** Patients’ Demographic at the Time of Diagnosis

	Absolute number	Relative (%)
Number of patients	62	100
Sex		
Female	24	38.7
Male	38	61.3
Mean age (years)		
Total (min.–max.)	64 (41–84)	
Female	66 (52–79)	
Male	63 (41–84)	

**Table 4:** Patients’ Clinical Characteristics at the Time of Diagnosis Including Age, TNM Stage Distribution, Type of Therapy, and Disease Course

	Absolute number	Relative (%)
T stage		
Tx	2	3.2
T0	0	0
T1	3	4.8
T2	16	25.8
T3	35	56.5
T4	6	9.7
N stage		
Nx	10	16.1
N0	5	8.1
N1	45	72.6
N2	2	3.2
M stage		
Mx	3	4.8
M0	26	41.9
M1	33	53.2
UICC-stadium		
IA	3	4.8
IB	2	3.2
IIA	0	0
IIB	16	25.8
III	5	8.1
IV	33	53.2
Unknown	3	4.8
Therapy		
Resection + chemotherapy	18	29.0
Resection only	5	8.1
Chemotherapy only	39	62.9
Disease course (n = 62)		
1 = no PD at t2	37	59.7
2 = PD at t2, no PD at t1	18	29.0
3 = PD at t2, PD at t1	7	11.3
Disease course (n = 35)		
1 = no PD at t3	16	45.7
2 = PD at t3, no PD at t2	13	37.1
3 = PD at t3, PD at t2	6	17.1

Additional information about patients’ therapy and disease course is provided. The third column shows the corresponding relative frequencies of patients. T: primary tumor; N: lymph nodes; M: metastasis; UICC: Union Internationale Contre le Cancer; PD: progressive disease.

**Table 5:** Characteristics and Measurements From t0 to t3

	t0		t1		t2		t3	
	Absolute	h (%)	Absolute	h (%)	Absolute	h (%)	Absolute	h (%)
Patients	62	100	56	90.3	62	100	35	56.5
PMI (cm^2^/m^2^), mean ± SD								
Total	5.45 ± 1.65		5.05 ± 1.45		4.96 ± 1.69		5.05 ± 1.52	
Female	4.22 ± 0.78		3.90 ± 0.89		3.62 ± 0.80		3.87 ± 0.64	
Male	6.22 ± 1.59		5.69 ± 1.31		5.81 ± 1.55		5.59 ± 1.50	
Sarcopenia according to lowest quartile								
Total	16	25.8*	14	25.0*	16	25.8*	9	25.7*
Female	6	25.0*	5	25.0*	6	25.0*	3	27.3*
Male	10	26.3*	9	25.0*	10	26.3*	6	25.0*
Sarcopenia according to reference values								
Total	19	30.6*	27	48.2*	31	50.0*	17	48.6*
Female	7	29.2*	10	50.0*	15	62.5*	5	45.5*
Male	12	31.6*	17	47.2*	16	42.1*	12	50.0*

The percentage values marked with (*) refer, for better comparability, to the subset of the respective category that contained values. PMI: psoas muscle index; SD: standard deviation.

**Figure 2:**
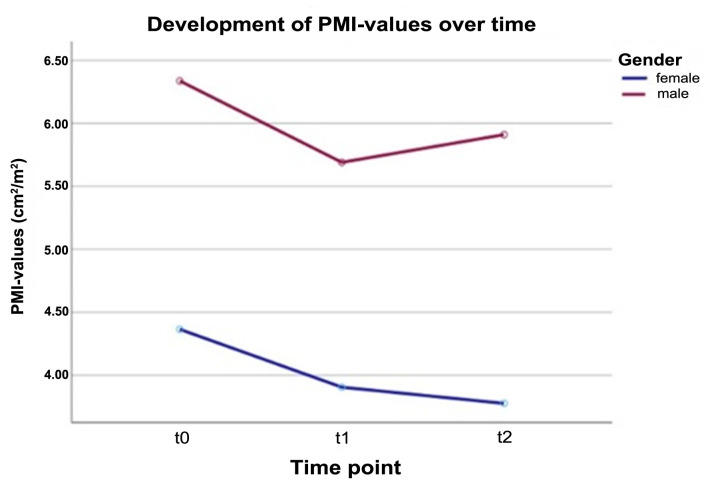
Sex-specific development of PMI values over time. As the number of evaluated patients decreased markedly at t3 (12 months), this follow-up time point is not shown. Values for all time points are provided in Table 5. PMI: psoas muscle index.

Sarcopenia was assessed longitudinally using the PMI based on both reference values and cohort-specific quartiles. Reference-based classification identified sarcopenia in 45.3% of patients. Across all time points, PMI values were consistently lower in women than men, reflecting persistent sex-specific differences (P < 0.005, partial η^2^ = 0.402). Overall, mean PMI declined from baseline (t0) to the first follow-up (t1) and remained relatively stable at t2, suggesting progressive muscle loss early after diagnosis without subsequent recovery (Fig. 2). This pattern was observed in both sexes, although absolute PMI values in men remained higher throughout follow-up. Contrast analyses revealed significant differences between t0 (M = 6.63, SD = 1.62) and t1 (M = 5.05, SD = 1.62), with a mean difference of −0.58 (SE = 0.113), Bonferroni-adjusted P < 0.001, partial η^2^ = 0.324. Similarly, the difference between t0 and t2 (M = 5.15, SD = 1.66) was also significant (Δ = −0.485, P < 0.001, partial η^2^ = 0.211). No significant difference was found between t1 and t2. For women, mean PMI values differed significantly across the three time points. Significant differences were observed between t0 (M = 4.36, SD = 0.75) and t1 (M = 3.90, SD = 0.89; P < 0.001, partial η^2^ = 0.456), as well as between t0 (M = 4.36, SD = 0.75) and t2 (M = 3.78, SD = 0.89; P < 0.001, partial η^2^ = 0.683). The difference between t1 and t2 was not statistically significant. For men, mean PMI values also showed significant differences across time. There was a significant decrease from t0 (M = 6.34, SD = 1.55) to t1 (M = 5.69, SD = 1.30; P < 0.001, partial η^2^ = 0.308), and a smaller but still significant difference between t0 (M = 6.34, SD = 1.55) and t2 (M = 5.91, SD = 1.52; P < 0.05, partial η^2^ = 0.126). However, the difference between t1 (M = 5.69, SD = 1.30) and t2 (M = 5.91, SD = 1.52) was not statistically significant.

Associations between sarcopenia and clinical variables were explored. No clear relationship was observed between sarcopenia prevalence and treatment type, including surgery with or without chemotherapy, indicating that sarcopenia occurred independently of therapeutic interventions. Chi-square tests showed no statistically significant association between surgery and sarcopenia status at any time point (e.g., t1: χ^2^(1) = 0.008, P > 0.05; t2: χ^2^(1) = 0.62, P > 0.05). Outcome analysis showed that using sex-specific quartiles for sarcopenia yielded largely comparable distributions between sarcopenic and non-sarcopenic patients at all time points. Chi-square tests confirmed no significant associations between sarcopenia and outcome. When reference-based thresholds were applied, a higher proportion of PD was observed in sarcopenic patients at t1 (χ^2^(2) = 6.55, P < 0.05, n = 56), although this pattern was limited by the sample size and was not consistent across other time points.

Radiomic analysis of the psoas major muscle enabled prediction of sarcopenia using machine learning models. Random forest consistently performed best, achieving an accuracy of 0.73 and ROC-AUC up to 0.81, with peak performance at t2. F_1_-scores ranged from 0.31 (decision tree) to 0.53 (random forest), with random forest achieving the highest F_1_ at t2 (0.75) (Table 6). Feature selection using LASSO regression identified the short axis and cross-sectional area of the psoas as the most relevant predictors, consistently across all time points, with the short axis peaking in relevance at t2 (23%) and remaining the strongest predictor at t3 (19%). For the analyses predicting sarcopenia diagnosis at time point t0, the short axis and the area emerged as the most relevant predictor variables, each contributing approximately 17%. At time point t1, these two variables remained the most relevant, with a contribution of about 20%. The same applied at time point t2; however, the relevance of the short axis increased to around 23%, while that of the area decreased to about 18%. At time point t3, the short axis continued to be the most relevant variable, contributing approximately 19%. In summary, the short axis and the area consistently emerged as the most important predictor variables, with their relevance peaking at measurement time point t2.

**Table 6:** ROC-AUC for the Three Prediction Algorithms

	t0	t1	t2	t3
Acc_M_rf	0.73 ± 0.05	0.76 ± 0.07	0.79 ± 0.12	0.72 ± 0.08
Acc_95% CI_rf	0.72–0.74	0.74–0.78	0.76–0.82	0.69–0.75
Auc_M_rf	0.81 ± 0.09	0.83 ± 0.07	0.84 ± 0.11	0.74 ± 0.14
Auc_95% CI_rf	0.79–0.83	0.81–0.85	0.81–0.87	0.69–0.79
F_1__M_rf	0.53 ± 0.21	0.70 ± 0.13	0.75 ± 0.06	0.71 ± 0.10
F_1__95% CI_rf	0.48–0.58	0.67–0.73	0.74–0.76	0.68–0.74
Acc_M_KNN	0.65 ± 0.08	0.45 ± 0.09	0.45 ± 0.08	0.47 ± 0.13
Acc_95% CI_KNN	0.63–0.67	0.43–0.47	0.43–0.47	0.43–0.51
Auc_M_KNN	0.58 ± 0.10	0.54 ± 0.09	0.54 ± 0.10	0.56 ± 0.20
Auc_95% CI_KNN	0.56–0.60	0.52–0.56	0.52–0.56	0.49–0.62
F_1__M_KNN	0.47 ± 0.28	0.53 ± 0.13	0.73 ± 0.09	0.59 ± 0.13
F_1__95% CI_KNN	0.40–0.54	0.50–0.56	0.71–0.75	0.55–0.63
Acc_M_dt	0.67 ± 0.07	0.66 ± 0.07	0.70 ± 0.05	0.76 ± 0.10
Acc_95% CI_dt	0.65–0.69	0.64–0.68	0.69–0.71	0.73–0.79
Auc_M_dt	0.68 ± 0.10	0.64 ± 0.07	0.72 ± 0.05	0.76 ± 0.10
Auc_95% CI_dt	0.66–0.70	0.62–0.66	0.71–0.73	0.73–0.79
F_1__M_dt	0.31 ± 0.12	0.71 ± 0.14	0.55 ± 0.10	0.54 ± 0.12
F_1__95% CI_dt	0.28–0.34	0.67–0.75	0.53–0.57	0.50–0.58

Acc: accuracy; CI: confidence interval; dt: decision tree; KNN: k-nearest neighbor; M: mean; rf: random forest; ROC-AUC: receiver operating characteristics area under the curve; SD: standard deviation.

**Figure 3:**
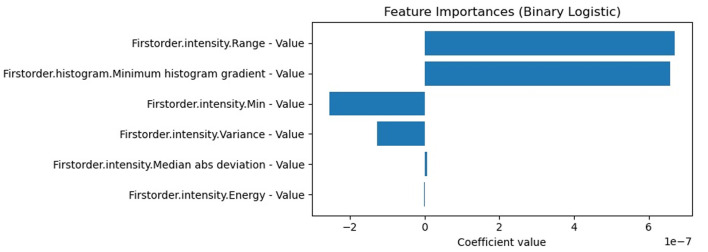
Selected features of the second logistic regression model (LogReg). Intensity range and minimum histogram gradient are the strongest predictors.

To assess prognostic potential, a two-step logistic regression model was trained on t0 radiomic features to predict outcomes during course of disease according to the ordinal scale on patient’s outcome from 1 to 3 (Table 4). Following feature selection, the reduced model achieved the best results for t3 with an accuracy of 0.850 (95% confidence interval (CI) 0.832–0.868), weighted F_1_ of 0.841 (95% CI 0.810–0.872), and AUC of 0.823 (95% CI 0.794–0.851) (sensitivity 0.625, specificity 1.000, precision/recall 1.000). Among selected features, intensity range and minimum histogram gradient were the strongest predictors, while other features had minimal impact (Fig. 3). Model reproducibility was confirmed through repeated experiments with random patient splits.

Interobserver agreement for psoas cross-sectional area measurements was very strong (r = 0.86), indicating good reproducibility and reliability of the radiological assessments.

## Discussion

The aim of this study was to retrospectively evaluate the presence and longitudinal progression of sarcopenia in patients with PDAC using CT-based imaging and clinical characteristics, and to assess its potential impact on PD.

### Imaging time points

The use of fixed CT imaging time points enabled a standardized longitudinal assessment under routine clinical conditions, reducing variability arising from differences in scan timing and ensuring that temporal muscle changes were evaluated at comparable stages of the disease and treatment course, thereby improving the reliability of within-patient comparisons. The inclusion criterion of at least three CT examinations introduces substantial survivor and selection bias. Patients experiencing rapid clinical deterioration, early death, or incomplete imaging follow-up were systematically excluded, resulting in an overrepresentation of individuals with more favorable disease trajectories. As a consequence, the observed longitudinal patterns of muscle loss and the association between sarcopenia and prognosis may be overestimated. Imaging findings at later time points primarily reflect a subgroup of patients with ongoing clinical stability rather than the broader PDAC population.

Despite this limitation, consistent imaging intervals strengthen internal validity and allow meaningful evaluation of longitudinal trends. Future studies integrating imaging with time-to-event analyses could further improve interpretability.

### Sarcopenia and influencing factors

Sarcopenia prevalence varied widely depending on the applied definition (25–62.5%) and increased over time. Women consistently showed lower PMI values than men. Using reference cut-offs derived from a healthy young population, 45.3% of patients were classified as sarcopenic, closely matching the prevalence reported by Haiducu et al in pancreatic cancer (approximately 45%) [11]. This aligns the EWGSOP2 recommendation to apply standardized reference values, enhancing comparability across studies [1]. Variability in reported sarcopenia prevalence is largely attributable to differences in diagnostic criteria and assessment methods [7, 16, 39–43]. Accordingly, two commonly used radiological definitions were evaluated. The quartile-based approach showed no clear association with disease progression, although a trend was observed in men at baseline. In contrast, reference-based sarcopenia demonstrated a modest association with PD at t1, though no statistically significant group differences were observed. Notably, PMI values declined consistently over time, suggesting that reduced muscle mass may be linked to PD. These findings underscore the importance of standardized sarcopenia definitions, particularly in small, heterogeneous cohorts.

### Radiological parameters

All muscle parameters were obtained from CT imaging, the standard modality for sarcopenia assessment in retrospective and opportunistic settings [44]. CT has been shown to provide more robust quantification of abdominal skeletal muscle mass than MRI, owing to superior anatomical delineation [45]. However, CT-based evaluation of muscle quality has inherent limitations.

Muscle density can be affected by factors such as hydration status, cardiac output, and regional perfusion [46]. While the contrast phase has minimal impact on muscle area, density values may vary between native and contrast-enhanced scans [46, 47]. To minimize such variability, only portal venous phase CT scans with standardized delay times and weight-adapted contrast dosing were included. This uniform acquisition protocol reduces phase-related bias, which is particularly important for radiomic features derived from Hounsfield unit intensity and texture.

### Assessment of sarcopenia

There is currently no universally accepted radiological definition of sarcopenia. In this study the choice of method was guided by its retrospective design and imaging feasibility. The PMI is a widely used surrogate for skeletal muscle mass but assumes uniform muscle distribution and may not capture asymmetries due to neurological or musculoskeletal conditions [48]. Muscle indices may be normalized to height^2^, weight, or BMI, with no consensus on a preferred approach recommended by the EWGSOP [1]. Alternative indices such as total abdominal muscle area include multiple muscle groups but were limited in this cohort due to postoperative changes affecting reproducibility. Of the 62 included patients, 23 underwent surgical resection. Among these, 20 exhibited postoperative changes in the rectus abdominis muscle that limited the assessment of total abdominal muscle area and precluded direct comparison with baseline CT scans. Therefore, the psoas muscle was selected as a consistent CT-based marker for the entire cohort, including patients with and without surgical intervention. Nonetheless, segmentation of all muscles at the L3 level was performed for radiomic analyses. Within these analyses, the rectus abdominis muscle showed a high degree of variability (data not shown).

While the PMI is a convenient and widely used measure in retrospective imaging studies, it represents only a fraction of total skeletal muscle mass and may not fully capture global sarcopenia. Compared to the SMI, which considers all major muscles at the L3 level, PMI may underestimate or misrepresent overall muscle depletion, particularly in patients with asymmetric or selective muscle loss. Nevertheless, PMI has been shown to correlate with clinical outcomes and provides a pragmatic surrogate in settings where full SMI assessment is not feasible [2, 4, 27]. In the present study reference-based PMI demonstrated prognostic relevance at baseline, whereas quartile-based definitions did not. LASSO regression further identified psoas short axis and muscle area as key morphological predictors. Combining reference-based PMI with these robust morphologic parameters may represent a hybrid radiological definition with enhanced prognostic performance, warranting future validation. A direct comparison between PMI and SMI would be particularly valuable for future validation and to improve the generalizability of our findings.

### Radiomics

Radiomic analysis revealed that intensity- and texture-based features provided the strongest prognostic information. Intensity range and minimum histogram gradient were the most relevant predictors for disease progression status after 12 months, whereas morphological parameters were most informative for sarcopenia classification. This finding suggests that muscle quality, heterogeneity, and fat infiltration may be more informative prognostic markers than muscle quantity alone. These are consistent with growing evidence highlighting the prognostic relevance of muscle density and myosteatosis in oncology, particularly in pancreatic cancer [49–52]. Muscle changes at the intermediate time point (t2) were especially informative, potentially reflecting more stable alteration patterns, in line with observations by Rizzo et al [20]. Prediction accuracy remained high at t3 despite a smaller cohort size, suggesting a homogenization of muscle changes in advanced disease, as previously reported [25, 53]. This may be attributable to reduced physical activity and increasing systemic inflammation [54]. Importantly, baseline radiomic features combined with machine learning showed an association with later disease progression, suggesting a potential role for radiomics in early risk stratification. However, these findings should be considered exploratory, as the prognostic model was developed in a limited sample without external validation or comparison to established clinical predictors. Therefore, the incremental value of radiomics beyond conventional factors such as disease stage, metastatic status, therapy type, performance status, or baseline PMI remains to be determined. Further studies in larger, independent cohorts, including direct comparisons with and integration of clinical models, are required before clinical applicability can be established. In addition, standardization of imaging acquisition, segmentation, and feature extraction remains essential to improve reproducibility and support future clinical translation [20].

### Limitations

This study is limited by its retrospective design, which restricts assessment to CT-based measures of muscle quantity and quality without functional evaluation, in contrast to the EWGSOP definition, which requires both muscle mass and function. The relatively small cohort size and heterogeneity in disease stage and treatment further increase the risk of overfitting and limit generalizability, despite the use of cross-validation and repeated random splits. This study is also limited by the use of fixed CT follow-up time points, which may result in selection bias due to the exclusion of patients who experienced disease progression or death between scheduled scans. Consequently, imaging findings at later time points may overrepresent patients with favorable clinical courses and may not be fully generalizable to the entire treated population. Furthermore, patient outcomes and PD status were defined solely according to RECIST criteria. In PDAC, however, disease progression is often complex, involving non-measurable lesions, peritoneal disease, clinical deterioration, biochemical markers, and multidisciplinary team (MDT) assessments, which are not fully captured by RECIST measurements alone. In this study, PD was defined purely radiologically according to RECIST, as this remains the only viable approach for retrospective analyses of sarcopenia and ensures comparability with previous studies using similar definitions. Future prospective studies should integrate functional measures, inflammatory markers, and multidisciplinary clinical parameters to more comprehensively assess the prognostic role of sarcopenia.

## Data Availability

The data that support the findings of this study are available from the corresponding author upon reasonable request.

## Abbreviations

AI: artificial intelligence
BMI: body mass index
CR: complete remission
CT: computed tomography
EWGSOP: European Working Group on Sarcopenia in Older People
KNN: k-nearest neighbors
L: lumbar level
LASSO: least absolute shrinkage and selection operator
LD: longest diameter
MDT: multidisciplinary team
ML: machine learning
MRI: magnetic resonance imaging
PACS: picture archiving and communication system
PD: progressive disease
PDAC: pancreatic ductal adenocarcinoma
PMI: psoas muscle index
PR: partial remission
RECIST: Response Evaluation Criteria in Solid Tumors
RIS: radiology information system
ROC-AUC: receiver operating characteristic area under the curve
ROI: region of interest
SD: standard deviation
SMA: skeletal muscle area
SMD: skeletal muscle density
SMI: skeletal muscle index
SOD: sum of diameters
TAMA: total abdominal muscle area
UICC: Union Internationale Contre le Cancer

## References

[1] Cruz-Jentoft AJ, Bahat G, Bauer J, Boirie Y, Bruyere O, Cederholm T, Cooper C (2019). Sarcopenia: revised European consensus on definition and diagnosis. Age Ageing.

[2] Ritz A, Kolorz J, Hubertus J, Ley-Zaporozhan J, von Schweinitz D, Koletzko S, Haberle B (2021). Sarcopenia is a prognostic outcome marker in children with high-risk hepatoblastoma. Pediatr Blood Cancer.

[3] Triarico S, Rinninella E, Mele MC, Cintoni M, Attina G, Ruggiero A (2022). Prognostic impact of sarcopenia in children with cancer: a focus on the psoas muscle area (PMA) imaging in the clinical practice. Eur J Clin Nutr.

[4] Ritz A, Froeba-Pohl A, Kolorz J, Vigodski V, Hubertus J, Ley-Zaporozhan J, von Schweinitz D (2021). Total psoas muscle area as a marker for sarcopenia is related to outcome in children with neuroblastoma. Front Surg.

[5] Nipp RD, Fuchs G, El-Jawahri A, Mario J, Troschel FM, Greer JA, Gallagher ER (2018). Sarcopenia is associated with quality of life and depression in patients with advanced cancer. Oncologist.

[6] Pamoukdjian F, Bouillet T, Levy V, Soussan M, Zelek L, Paillaud E (2018). Prevalence and predictive value of pre-therapeutic sarcopenia in cancer patients: a systematic review. Clin Nutr.

[7] Peng P, Hyder O, Firoozmand A, Kneuertz P, Schulick RD, Huang D, Makary M (2012). Impact of sarcopenia on outcomes following resection of pancreatic adenocarcinoma. J Gastrointest Surg.

[8] Leeper CM, Lin E, Hoffman M, Fombona A, Zhou T, Kutcher M, Rosengart M (2016). Computed tomography abbreviated assessment of sarcopenia following trauma: The CAAST measurement predicts 6-month mortality in older adult trauma patients. J Trauma Acute Care Surg.

[9] Xu J, Zheng B, Zhang S, Zeng T, Chen H, Zheng W, Chen C (2019). Effects of preoperative sarcopenia on postoperative complications of minimally invasive oesophagectomy for oesophageal squamous cell carcinoma. J Thorac Dis.

[10] Zwart AT, van der Hoorn A, van Ooijen PMA, Steenbakkers R, de Bock GH, Halmos GB (2019). CT-measured skeletal muscle mass used to assess frailty in patients with head and neck cancer. J Cachexia Sarcopenia Muscle.

[11] Haiducu C, Buzea A, Mirea LE, Dan GA (2021). The prevalence and the impact of sarcopenia in digestive cancers. A systematic review. Rom J Intern Med.

[12] https://www.krebsdaten.de/Krebs/DE/Content/Krebsarten/Bauchspeicheldruesenkrebs/bauchspeicheldruesenkrebs_node.html.

[13] Hezel AF, Kimmelman AC, Stanger BZ, Bardeesy N, Depinho RA (2006). Genetics and biology of pancreatic ductal adenocarcinoma. Genes Dev.

[14] McGuigan A, Kelly P, Turkington RC, Jones C, Coleman HG, McCain RS (2018). Pancreatic cancer: a review of clinical diagnosis, epidemiology, treatment and outcomes. World J Gastroenterol.

[15] Cai J, Chen H, Lu M, Zhang Y, Lu B, You L, Zhang T (2021). Advances in the epidemiology of pancreatic cancer: Trends, risk factors, screening, and prognosis. Cancer Lett.

[16] Grotenhuis BA, Shapiro J, van Adrichem S, de Vries M, Koek M, Wijnhoven BP, van Lanschot JJ (2016). Sarcopenia/muscle mass is not a prognostic factor for short- and long-term outcome after esophagectomy for cancer. World J Surg.

[17] Shachar SS, Williams GR, Muss HB, Nishijima TF (2016). Prognostic value of sarcopenia in adults with solid tumours: A meta-analysis and systematic review. Eur J Cancer.

[18] Kim IH, Choi MH, Lee IS, Hong TH, Lee MA (2021). Clinical significance of skeletal muscle density and sarcopenia in patients with pancreatic cancer undergoing first-line chemotherapy: a retrospective observational study. BMC Cancer.

[19] Cespedes Feliciano EM, Avrutin E, Caan BJ, Boroian A, Mourtzakis M (2018). Screening for low muscularity in colorectal cancer patients: a valid, clinic-friendly approach that predicts mortality. J Cachexia Sarcopenia Muscle.

[20] Rizzo S, Botta F, Raimondi S, Origgi D, Fanciullo C, Morganti AG, Bellomi M (2018). Radiomics: the facts and the challenges of image analysis. Eur Radiol Exp.

[21] Lambin P, Leijenaar RTH, Deist TM, Peerlings J, de Jong EEC, van Timmeren J, Sanduleanu S (2017). Radiomics: the bridge between medical imaging and personalized medicine. Nat Rev Clin Oncol.

[22] Chen G, Fan X, Wang T, Zhang E, Shao J, Chen S, Zhang D (2023). A machine learning model based on MRI for the preoperative prediction of bladder cancer invasion depth. Eur Radiol.

[23] Zwanenburg A, Vallieres M, Abdalah MA, Aerts H, Andrearczyk V, Apte A, Ashrafinia S (2020). The image biomarker standardization initiative: standardized quantitative radiomics for high-throughput image-based phenotyping. Radiology.

[24] Ge G, Zhang J (2023). Feature selection methods and predictive models in CT lung cancer radiomics. J Appl Clin Med Phys.

[25] Vogele D, Mueller T, Wolf D, Otto S, Manoj S, Goetz M, Ettrich TJ (2024). Applicability of the CT radiomics of skeletal muscle and machine learning for the detection of sarcopenia and prognostic assessment of disease progression in patients with gastric and esophageal tumors. Diagnostics (Basel).

[26] Eisenhauer EA, Therasse P, Bogaerts J, Schwartz LH, Sargent D, Ford R, Dancey J (2009). New response evaluation criteria in solid tumours: revised RECIST guideline (version 1.1). Eur J Cancer.

[27] Fitzpatrick JA, Basty N, Cule M, Liu Y, Bell JD, Thomas EL, Whitcher B (2020). Large-scale analysis of iliopsoas muscle volumes in the UK Biobank. Sci Rep.

[28] Bahat G, Turkmen BO, Aliyev S, Catikkas NM, Bakir B, Karan MA (2021). Cut-off values of skeletal muscle index and psoas muscle index at L3 vertebra level by computerized tomography to assess low muscle mass. Clin Nutr.

[29] Taguchi S, Akamatsu N, Nakagawa T, Gonoi W, Kanatani A, Miyazaki H, Fujimura T (2016). Sarcopenia evaluated using the skeletal muscle index is a significant prognostic factor for metastatic urothelial carcinoma. Clin Genitourin Cancer.

[30] Castillo-Angeles M, Uyeda JW, Seshadri AJ, Ramsis R, Okafor BU, Nitzschke S, Rangel EL (2022). Sarcopenia is associated with increased mortality in patients with necrotizing soft tissue infections. J Surg Res.

[31] Cao Q, Xiong Y, Zhong Z, Ye Q (2019). Computed tomography-assessed sarcopenia indexes predict major complications following surgery for hepatopancreatobiliary malignancy: a meta-analysis. Ann Nutr Metab.

[32] Rangel EL, Rios-Diaz AJ, Uyeda JW, Castillo-Angeles M, Cooper Z, Olufajo OA, Salim A (2017). Sarcopenia increases risk of long-term mortality in elderly patients undergoing emergency abdominal surgery. J Trauma Acute Care Surg.

[33] Bailey CM, Schaverien MV, Garvey PB, Liu J, Butler CE, Mericli AF (2020). The impact of sarcopenia on oncologic abdominal wall reconstruction. J Surg Oncol.

[34] Harris CR, Millman KJ, van der Walt SJ, Gommers R, Virtanen P, Cournapeau D, Wieser E (2020). Array programming with NumPy. Nature.

[35] Buitinck L, Louppe G, Blondel M

[36] Guillaume Lemaître FN, Christos K (2017). Aridas. Imbalanced-learn: A Python Toolbox to Tackle the Curse of Imbalanced Datasets in Machine Learning. Journal of Machine Learning Research.

[39] Batsis JA, Barre LK, Mackenzie TA, Pratt SI, Lopez-Jimenez F, Bartels SJ (2013). Variation in the prevalence of sarcopenia and sarcopenic obesity in older adults associated with different research definitions: dual-energy X-ray absorptiometry data from the National Health and Nutrition Examination Survey 1999-2004. J Am Geriatr Soc.

[40] Bijlsma AY, Meskers CG, Ling CH, Narici M, Kurrle SE, Cameron ID, Westendorp RG (2013). Defining sarcopenia: the impact of different diagnostic criteria on the prevalence of sarcopenia in a large middle aged cohort. Age (Dordr).

[41] Naumann P, Eberlein J, Farnia B, Hackert T, Debus J, Combs SE (2019). Continued weight loss and sarcopenia predict poor outcomes in locally advanced pancreatic cancer treated with chemoradiation. Cancers (Basel).

[42] Dolan DR, Knight KA, Maguire S, Moug SJ (2019). The relationship between sarcopenia and survival at 1 year in patients having elective colorectal cancer surgery. Tech Coloproctol.

[43] Choi MH, Oh SN, Lee IK, Oh ST, Won DD (2018). Sarcopenia is negatively associated with long-term outcomes in locally advanced rectal cancer. J Cachexia Sarcopenia Muscle.

[44] Beaudart C, McCloskey E, Bruyere O, Cesari M, Rolland Y, Rizzoli R, Araujo de Carvalho I (2016). Sarcopenia in daily practice: assessment and management. BMC Geriatr.

[45] Park J, Gil JR, Shin Y, Won SE, Huh J, You MW, Park HJ (2019). Reliable and robust method for abdominal muscle mass quantification using CT/MRI: An explorative study in healthy subjects. PLoS One.

[46] Rollins KE, Gopinath A, Awwad A, Macdonald IA, Lobo DN (2020). Computed tomography-based psoas skeletal muscle area and radiodensity are poor sentinels for whole L3 skeletal muscle values. Clin Nutr.

[47] Derstine BA, Holcombe SA, Goulson RL, Ross BE, Wang NC, Sullivan JA, Su GL (2017). Quantifying sarcopenia reference values using lumbar and thoracic muscle areas in a healthy population. J Nutr Health Aging.

[48] Shen W, Punyanitya M, Wang Z, Gallagher D, St-Onge MP, Albu J, Heymsfield SB (2004). Total body skeletal muscle and adipose tissue volumes: estimation from a single abdominal cross-sectional image. J Appl Physiol (1985).

[49] Perrier M, Fontaine M, Bertin E, Carlier C, Botsen D, Djelouah M, Francois E (2025). Impact of low muscle mass and myosteatosis on treatment toxicity and survival outcomes in non-resectable pancreatic cancer patients treated with chemoradiotherapy. Eur J Clin Nutr.

[50] Nowak S, Kloth C, Theis M, Marinova M, Attenberger UI, Sprinkart AM, Luetkens JA (2024). Deep learning-based assessment of CT markers of sarcopenia and myosteatosis for outcome assessment in patients with advanced pancreatic cancer after high-intensity focused ultrasound treatment. Eur Radiol.

[51] Keyl J, Bucher A, Jungmann F, Hosch R, Ziller A, Armbruster R, Malkomes P (2024). Prognostic value of deep learning-derived body composition in advanced pancreatic cancer-a retrospective multicenter study. ESMO Open.

[52] Zhang X, Wei L, Li J, Deng Y, Xu W, Chen D, Li X (2024). Influence of myosteatosis on survival of patients with pancreatic cancer: a systematic review and meta-analysis. iScience.

[53] Yoon HG, Oh D, Ahn YC, Noh JM, Pyo H, Cho WK, Song YM (2020). Prognostic impact of sarcopenia and skeletal muscle loss during neoadjuvant chemoradiotherapy in esophageal cancer. Cancers (Basel).

[54] Warnberg J, Cunningham K, Romeo J, Marcos A (2010). Physical activity, exercise and low-grade systemic inflammation. Proc Nutr Soc.

